# Aging Affects the Mental Rotation of Left and Right Hands

**DOI:** 10.1371/journal.pone.0006714

**Published:** 2009-08-26

**Authors:** Arnaud Saimpont, Thierry Pozzo, Charalambos Papaxanthis

**Affiliations:** 1 INSERM U887 Motricité-Plasticité, Université de Bourgogne, Dijon, France; 2 Italian Institute of Technology, Genoa, Italy; University of Leuven, Belgium

## Abstract

**Background:**

Normal aging significantly influences motor and cognitive performance. Little is known about age-related changes in action simulation. Here, we investigated the influence of aging on implicit motor imagery.

**Methodology/Principal Findings:**

Twenty young (mean age: 23.9±2.8 years) and nineteen elderly (mean age: 78.3±4.5 years) subjects, all right-handed, were required to determine the laterality of hands presented in various positions. To do so, they mentally rotated their hands to match them with the hand-stimuli. We showed that: (1) elderly subjects were affected in their ability to implicitly simulate movements of the upper limbs, especially those requiring the largest amplitude of displacement and/or with strong biomechanical constraints; (2) this decline was greater for movements of the non-dominant arm than of the dominant arm.

**Conclusions/Significance:**

These results extend recent findings showing age-related alterations of the explicit side of motor imagery. They suggest that a general decline in action simulation occurs with normal aging, in particular for the non-dominant side of the body.

## Introduction

Motor imagery or internal action simulation can be defined as the ability to mentally simulate movements without actually executing them. The simulation theory, developed by Jeannerod and colleagues [1], postulates that simulated (covert) actions share common neurocognitive mechanisms with their executed (overt) counterparts. For example, at the behavioural level, psychophysical investigations have consistently shown that the time required to imagine a movement closely parallels the time necessary to physically execute it and that similar physical laws (e.g. Fitts law) apply to both covert and overt actions [2]–[4]. Furthermore, at the neural level, several neuroimaging studies have revealed that brain regions involved in action simulation partially overlap with those implicated in action execution. Indeed, it has been well-established that the posterior parietal cortex, the premotor, the supplementary and the primary motor areas, as well as the basal ganglia and the cerebellum, are activated during motor imagery and movement execution [1], [5].

Action simulation constitutes a more or less explicit process depending on how it is triggered [1], [6]. When people voluntarily decide to imagine movements, the process is explicit. This is, for instance, the case when athletes mentally prepare themselves before a competition. Alternatively, when people have to make prospective action judgments, that is to say estimations about how they would perform actions or about the feasibility of actions (without executing them), it has been shown that they implicitly simulate these actions [7]. Motor imagery is also implicitly triggered when subjects are engaged in the “hand laterality” task, initially developed by Cooper and Shepard [8] and later popularized by Parsons [9], [10]. In this task, participants have to determine the laterality of images of left and right hands presented in different orientations. Usually, they solve this problem by mentally rotating their own hands from their current position to the orientation of the stimuli for comparison. Indeed, response times in handedness recognition are highly correlated to the execution times required to physically match subjects' hands with the stimuli and increase with the length of the hand trajectories as well as the biomechanical constraints normally applied during the executed movements [9], [10].

Interestingly, this type of implicit tasks has been widely used to study action simulation in several clinical populations [6]. For example, it has been shown that this process is affected in brain-damaged individuals [11], people suffering from peripheral nerve injuries [12], patients with chronic arm pain [13], or people with Parkinson's disease [14]. Curiously, however, there are, to our knowledge, no data regarding the evolution of implicit motor imagery with normal aging. This could be an interesting issue since the brain undergoes considerable changes with advancing age, such as shrinkage of grey matter volume and white matter loss [15] as well as neural and functional reorganizations [16]. These neural modifications often have behavioural consequences and elderly people perform differently and often worse than younger ones in various cognitive and sensorimotor tasks [17], [18]. For example, visual mental rotation studies have revealed that elderly subjects are slower and less accurate than younger adults in identifying objects presented in different orientations [19], [20]. Furthermore, age-related alterations have been observed in sensorimotor tasks [21] and recent studies have shown that explicit motor imagery is affected in elderly individuals for movements requiring high spatiotemporal [22] or dynamic [23] control.

The general purpose of this experiment was to study the influence of normal aging on the implicit mental simulation of upper-limb movements through a simplified version of the “hand laterality” task. We chose this task because it allows an increase in the difficulty of the simulated movements simply by manipulating the orientation of the visual stimuli. We made two main predictions. First, given previous research on the relative age-related decline of explicit motor imagery [22], [23] and the neurocognitive similarities existing between the explicit and implicit sides of action simulation [24], we expected that elderly subjects would be less efficient than their younger counterparts in implicitly simulating upper-limb movements. Second, in these types of tasks, young right-handed subjects are often better at mentally moving their dominant arm than their non-dominant arm [12], [25]. Moreover, it has been shown that the left hand is more affected by age (than the right hand) in the execution of different motor tasks [26], and a recent study has revealed a more prominent age-related decline for the non-dominant arm when subjects had to explicitly simulate pointing movements [27]. Thus, we anticipated that, compared to their younger counterparts, elderly subjects would be even less efficient in implicitly simulating left upper-limb movements than right upper-limb movements.

## Methods

### Participants

Twenty young and twenty two elderly adults initially participated in the study. After the elimination of three aged participants who failed to accomplish the task (see Procedure and Data analysis for details), twenty young (mean age 23.9±2.8 years, range 20–30, eleven females) and nineteen old (mean age 78.3±4.5 years, range 75–87, twelve females) subjects were finally included in the experiment. The young adults were students from the University of Bourgogne. The elderly adults were pensioners recruited by a local newspaper. They engaged in regular physical activity (about two days per week) and had at least one cognitive activity per day (e.g. reading newspapers, books or doing crosswords). All participants were consistent right-handers (without a history of hand switching during their lifetime) as measured by the Edinburgh Handedness Inventory [28]. The average index of laterality was 0.89±0.07 (range 0.75–1.00) for the young adults and 0.92±0.09 (range 0.70–1.00) for the elderly adults. All subjects were in good health, with normal or corrected-to-normal vision and had no history of motor or neurological disorders as assessed by a brief questionnaire. A French version of the mini mental state of examination (MMSE) [29] was administered to assess global cognitive function of the elderly subjects. None of them had cognitive impairment (mean score = 29.1±1.0, range 27–30). Visuospatial span for both groups was also assessed with the Corsi block-tapping task [30]. All subjects had values above the means of their range of age (young: 7.5±0.7, old: 5.6±0.9). The simple (visual) reaction time (SRT) for the left and right hand was also measured (young: left = 286±55 ms, right = 283±38 ms; old: left = 246±40, right = 244±35). The characteristics of the two age groups are summarized in Table 1.

**Table 1:** Characteristics of the two groups of subjects.

Group	Old (n = 19)	Young (n = 20)
Sex (M/F)	7/12	9/11
Age (years)	78.3±4.5	23.9±2.8
Handedness score^1^	0.92±0.09	0.89±0.07
MMSE score	29.1±1.0	X
Visuospatial span^2^	5.6±0.9	7.5±0.7
Simple reaction time (ms)	Left hand: 286±55	Left hand: 246±40
	Right hand: 283±38	Right hand: 244±35

Plus-minus values are means±SD.

1 Edimburgh inventory score.

2 Corsi block-tapping task.

All participants gave their written informed consent prior to their inclusion in this study, which was approved by the Dijon Regional Ethics Committee.

### Stimuli

**Figure 1:**
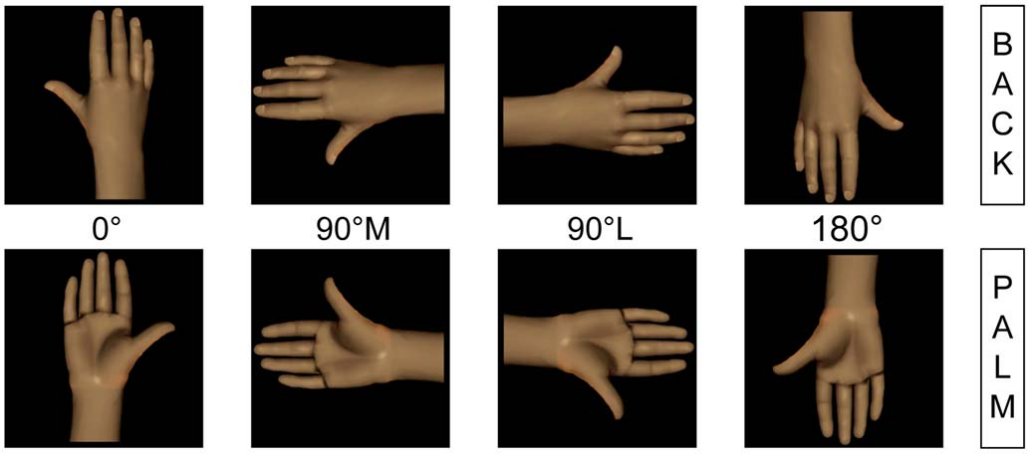
Right-hand stimuli. Right-hand stimuli in back and palm views at orientations of 0°, 90° medial (90°M), 90° lateral (90°L) and 180°.

The stimuli used in the current study were depictions of realistic left and right hands (Figure 1) rendered with Poser 4.0 software (Curious Labs, Santa Cruz, CA, USA). The hands could be seen from two perspectives (palm and back) and in four different orientations in the picture plane: 0° = facing up, 90° medial (90°M) = facing towards the participant's midsagittal plane, 90° lateral (90°L) = facing away from the participant's midsagittal plane, 180° = facing down.

Left and right hands were mirror images of each other in order to ensure that each stimulus was identical except for the change in position. Thus, 16 different stimuli (2 hands×2 views×4 orientations) were created. Each hand-picture was presented individually on a black background and was 15 cm in height and 10 cm in width. Stimuli were displayed on a laptop computer by means of specific software developed in our laboratory, which also recorded the response time (i.e., from stimulus onset to button-press; temporal resolution of 1 ms) as well as the accuracy of each response of the subjects.

### Procedure

All subjects first filled out the health questionnaire, then underwent the SRT test, and finally the Corsi block-tapping task. For the SRT test, subjects were seated in front of a laptop computer with their left (right) index finger placed on the left (right) button of the touchpad. They were told to press the button as soon as a little square appeared in the middle of the screen. A block of 20 trials (with an intertrial interval varying randomly between 1500 and 2000 ms) was performed for each index finger. The SRT for each hand was thus calculated as the mean of those trials. The Corsi apparatus consisted of nine blocks arranged irregularly on a board. The blocks were tapped by the experimenter in randomized sequences of increasing length. Immediately after each tapped sequence, the participants attempted to reproduce it, progressing until it was not possible. The participants had a maximum of two trials by level of difficulty and their visuospatial memory span was assessed as the maximum number of blocks correctly recalled at least once. Aged subjects additionally completed the MMSE.

Then, all subjects participated in a training session which was divided into two phases. The first phase was designed to ensure that all participants - more particularly the older ones - were actually able to move their upper-limbs in the different configurations imposed by the hand-stimuli. Subjects were seated on a chair, with their hands resting palm-down on the keyboard of a laptop computer placed on a table in front of them. They were shown the 16 different stimuli (2 hands×2 views×4 orientations) on the computer screen, one at a time, in random order. They were asked to physically move and superimpose the hand corresponding to each stimulus. The experimenter controlled the presentation of the successive stimuli with a computer mouse. All participants were able to match their hands with the different stimuli (although some of the elderly subjects sometimes hesitated with the most unnatural hand-postures).

The second phase of the training session was designed to familiarize the subjects with the experimental protocol. They were shown a sequence of 32 hand positions (the 16 different stimuli shown twice) on the computer screen. The stimuli followed each other in a pseudo-random order with the restriction that the same hand (regardless of the view and orientation it was presented) could not occur more than 4 times consecutively. The stimuli were interspersed with a white fixation cross (displayed for 2000 ms) and remained on the screen until subjects responded. Participants' hands rested palm down on the keyboard of the laptop computer. Their left index finger was placed on the left button of the touchpad and inversely for their right index finger. Vision of both their hands and forearms was prevented by a covering box. To respond, they had to press the left button for a left hand-stimulus and the right button for a right hand-stimulus. Otherwise, they were told to refrain from moving their head and hands during the presentation of the stimuli. No constraints of time were given during this training phase. All subjects correctly understood the instructions, but some of the elderly participants found it difficult to perform the task when the stimuli were presented in the most unusual positions. Subjects who failed to achieve (overall) at least 60% of correct responses were given additional practice. It was not necessary to provide more than 48 trials to each of the subjects except for one elderly participant who was finally removed from the experiment.

When the practice trials were completed, participants started the experimental session proper. The experiment was divided into 6 series, each series consisting of 32 stimuli (2×2 hands×2 views×4 orientations) presented in a random order except that the same hand could not appear more than 4 times in succession. Moreover, since the trials were divided into series, the same stimulus could not appear more than twice consecutively. A total of 192 trials were therefore administered to each participant. Between series, participants were allowed a small break (less than 1 min). As in the training session, participants were seated in front of the laptop with their hands resting palm-down on the keyboard (left finger on the left touchpad button, right finger on the right button). The trials were interspersed with a white fixation cross (displayed for 2000 ms) and remained on the screen until participants indicated their laterality by pressing either the left or the right button of the touchpad. Importantly, in this testing phase, subjects were told to respond as quickly and accurately as possible.

### Data analysis

Mean accuracy and response time (RT) were calculated for each participant within each cell (defined by hand, view and orientation). Accuracy was defined as the proportion of correct responses; RT corresponded to the interval between the onset of a stimulus and the push on one of the response buttons. Individual performance was considered above chance level when the overall number of correct responses was above 110/192 trials (one-tailed binomial test, *p*<0.005). Two older subjects (One male and one female) did not reach this criterion and their data were discarded from further analysis. For the calculation of RTs, only data from correct responses were included. RT outliers were excluded from analyses (see Results section). Precisely, we discarded RTs>8000 ms or RTs that exceeded the cell mean by at least two standard deviations [31]. Mean proportions of correct responses and RTs (log-transformed to reduce skewness) were analyzed with repeated-measures analyses of variance (ANOVAs). We conducted separate analyses of data in each view (back and palm). Each ANOVA had one between-subjects factor: Age (young, old), and two within-subjects factors: Hand (left, right) and Orientation (0°, 90°M, 90°L, 180°). Planned comparisons (two-tailed t-tests with Bonferroni correction for multiple comparisons, corrected *p* = 0.008) were conducted as appropriate. All analyses were performed using Statistica 6 (Statsoft, Tulsa, OK, USA).

## Results

### Correct responses

**Figure 2:**
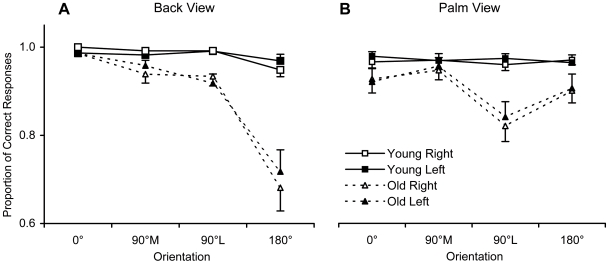
Accuracy. Mean proportions of correct responses (error bars represent standard errors) in the two groups, for hands shown in the four orientations, in back (A) and palm (B) views.

Proportions of correct responses for both groups are plotted separately for back and palm views in Figure 2A and B.

For the back view, older participants were less accurate than younger ones, *F*(1,37) = 45.08, *p*<0.0001, *η_p_^2^* = 0.55. There was also a main effect of Orientation, *F*(3,111) = 42.99, *p*<0.0001, *η_p_^2^* = 0.54. Planned comparisons indicated that the proportion of correct responses for the 180° stimulus was significantly lower compared to the other three stimuli (all *p*<0.0001) and that the accuracy for the 90°L and 90°M stimuli was lower than for the 0° stimulus (both *p*<0.005). The interaction between Orientation and Age was also significant, *F*(3,111) = 26.28, *p*<0.0001, *η_p_^2^* = 0.42. Planned comparisons revealed that, whereas the accuracy did not significantly differ between each orientation in the young group, fewer correct responses were given at 180° compared to the other orientations (all *p*<0.0001) and at 90°L and 90°M in comparison to 0° (both *p*<0.005) in the old group. No other main effects or interactions were significant. In particular, the non-significant effect of Hand, *F*(1,37)<1, and interaction between Hand and Age, *F*(1,37)<1, suggest that subjects of both groups did not favor their dominant hand compared to their non-dominant hand.

Similar results were found for the palm view. The ANOVA revealed significant main effects of Age, *F*(1,37) = 14.53, *p*<0.0005, *η_p_^2^* = 0.28, and Orientation, *F*(3,111) = 4.73, *p*<0.005, *η_p_^2^* = 0.11. For this latter effect, planned comparisons indicated that the accuracy for the 90°L stimulus was significantly lower than for the others. The interaction between Orientation and Age was also significant, *F*(3,111) = 4.17, *p*<0.01, *η_p_^2^* = 0.10. Planned comparisons showed that: (i) the accuracy was not significantly different between each orientation for younger subjects; (ii) the proportion of correct responses was significantly lower at 90°L compared to the other orientations for older subjects (all *p*<0.008). Here too, the effect of Hand, *F*(1,37)<1, and the Hand × Age interaction, *F*(1,37)<1, were not significant, indicating that participants of both groups did not favor their dominant hand.

### Response times

**Figure 3:**
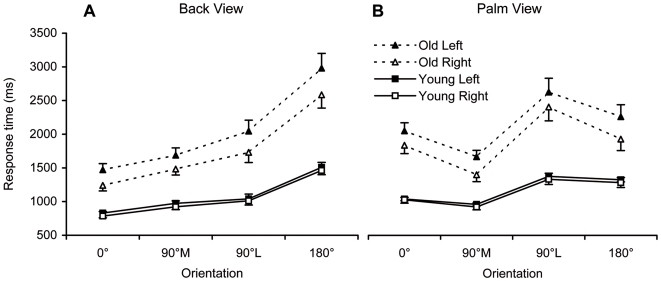
Response time. Mean response times (error bars represent standard errors) in the two groups, for hands shown in the four orientations, in back (A) and palm (B) views.

RT outliers were eliminated prior to analysis, resulting in the removal of 4.7% of all trials for the young participants and 6.9% for the older ones. For both groups, outliers were distributed equally across left and right stimuli, but for the older group, they were more likely to occur with the palm 90°L and back 180° conditions. Mean response times for both groups are plotted for back and palm views in Figure 3A and B.

For the back view, the ANOVA showed a main effect of Age, *F*(1,37) = 77.43, *p*<0.0001, *η_p_^2^* = 0.68, indicating that older adults were much slower than their younger counterparts. The Hand factor was also significant, *F*(1,37) = 21.09, *p*<0.0001, *η_p_^2^* = 0.36, revealing slower RTs for the left than the right hand stimuli. The analysis also showed a significant interaction between Age and Hand, *F*(1,37) = 7.33, *p*<0.01, *η_p_^2^* = 0.16, insofar as the difference in RT to discriminate non-dominant from dominant hands increased with age. There was a significant main effect of Orientation, *F*(3,111) = 108.63, *p*<0.0001, *η_p_^2^* = 0.75. Planned comparisons indicated that RTs were significantly higher at 180° compared to all other orientations as well at 90°L and 90°M compared to 0° (all *p*<0.0001). The interaction between Orientation and Age was also significant, *F*(3,111) = 5.00, *p*<0.005, *η_p_^2^* = 0.12. Planned comparisons revealed that the increase in RT between the 0° and 180° orientations, as well as between the 90°M and 180°M were significantly greater in the old group than in the younger one (both *p*<0.008).

Similar results were found for the palm view. The ANOVA showed significant main effects of Age, *F*(1,37) = 65.88, *p*<0.0001, *η_p_^2^* = 0.64, and Hand, *F*(1,37) = 11.16, *p*<0.005, *η_p_^2^* = 0.23, as well as a significant Age × Hand interaction, *F*(1,37) = 5.89, *p*<0.05, *η_p_^2^* = 0.14, indicating that the disadvantage in RT to identify right from left hand stimuli was greater for the elderly subjects. There was also a main effect of Orientation, *F*(3,111) = 48.10, *p*<0.0001, *η_p_^2^* = 0.57. Planned comparisons revealed that RTs were significantly longer at 90°L than at 90°M and 0°, as well as at 180° and 0° compared to 90°M (all *p*<0.0001). The interaction between Orientation and Age was also significant, *F*(3,111) = 4.65, *p*<0.005, *η_p_^2^* = 0.11. Planned comparisons indicated that the difference in RT between the 90°M and 90°L conditions, as well as between the 90°M and 0°M was significantly more pronounced for older subjects than for their younger counterparts (both *p*<0.005).

Finally, correlations between proportions of correct responses and RTs were calculated within both age groups to look for any speed-accuracy trade-off. In both groups, accuracy was negatively correlated with RTs (young: r = −0.32, *p*<0.01; old: r = −0.58, *p*<0.01). Correctness of decisions was thus associated with faster rather than slower responses, indicating that RT data cannot be explained by a strategy that sacrifices accuracy for speed.

## Discussion

The aim of the present study was to investigate the influence of normal aging on the implicit motor imagery of upper-limb movements, by means of the “hand laterality” task in which participants are required to discriminate between left and right hand-stimuli presented in various positions. The nature of the representations underlying imagery (analog/non-analog) has been, and is still, a matter of debate [32]. However, it is generally admitted that people solve the “hand laterality” task by mentally moving their own hands to match them with the different stimuli, as reflected by RTs that are highly correlated with execution times required to physically perform these movements [9], [10].

In the present study, in both age groups, the RTs profiles corroborate those usually found, with the longest RTs occurring at different orientations for back and palm views [9], [10], [13], [33]. This suggests that our subjects were mentally rotating their own hands to solve the task. Indeed, RTs increased as (i) the angular distance between the positions of the subjects' hands (i.e. back 0°) and the hand-stimuli increased (e.g. back 90°M/back 0°), (ii) the arm joint constraints normally applied during rotations of the hands were strong (e.g. palm 90°L/palm 90°M), and (iii) both angular distance and biomechanical constraints were important (back 180°).

For our elderly subjects, we found that: (i) they were affected in their ability to mentally simulate upper-limb movements, especially those requiring the largest amplitude of displacement or/and with strong biomechanical constraints (back 180° and palm 90°L), and (ii) this decline was greater for movements of the non-dominant than the dominant arm. Note that, compared to their younger counterparts, older adults were also particularly slow to recognize the palm 0° stimuli. This finding is not easily interpretable solely in terms of joint constraints or amplitude of displacement. The palm 0° stimuli are the direct mirror form of the back 0° stimuli, which are the most familiar and whose matching with subjects' hands is quite direct. It is thus possible that the visual familiarity between the two may have disturbed the subjects (and contributed to the increase in RTs), especially the elderly adults.

### General decline in action simulation with aging

Our findings showed that elderly subjects were less accurate and slower than their younger counterparts in their left-right hand judgments, and were particularly impaired in the most difficult conditions.

These results can be compared with previous findings which revealed that normal aging influences the ability to manipulate visual-mental images. Indeed, by using classic mental rotation tasks, in which subjects must identify objects (alphanumeric characters, geometric forms, etc.) presented in different orientations, numerous studies have shown that elderly people perform worse than younger ones, both in terms of RT and accuracy [19], [20], [34]. Furthermore, they may sometimes be severely impaired in their ability to rotate objects when the amount of mental rotation is too high [35].

The performances of our elderly subjects are thus comparable to those found in previous mental rotation studies using other types of stimuli. However, hand-pictures constitute a special class of stimuli in mental rotation tasks since they elicit motor imagery rather than visual imagery [36]. In other words, hand-pictures trigger the use of an internal (subject-centred) strategy, that is to say a mental rotation of ones own hands, whereas the other type of objects (non-corporeal) usually trigger the use of an external (object-centred) strategy, i.e. a mental transformation of the objects as if they are displaced by external forces [37]. Whereas these two types of strategies activate posterior parietal areas (involved in spatial transformations), only the internal strategy recruits motor regions in the precentral cortex [38], [39].

Thus, our findings extend those of previous studies by showing that subject-centred mental transformations, which involve visuospatial and motor processes, are affected by aging in a similar manner as object-centred mental transformations, which mainly involves visuospatial processes.

It is also of interest that the present results corroborate those of previous work on age-related changes in the explicit side of motor imagery. Indeed, using mental chronometry, Skoura et al. [22] and Personnier et al. [23] showed that elderly people were impaired in mentally simulating actions that required high spatiotemporal or dynamic control. For example, Skoura et al. [22] used a Fitts' paradigm in which participants had to physically move or to imagine moving their arm between two targets of varying size. Whereas young and elderly subjects obeyed Fitt's law when they executed the movements, insofar as they progressively slowed down as the size of the targets decreased, only young subjects showed the same pattern of responses when they imagined these movements. The temporal dissimilarities between overt and covert movements in elderly subjects when the task' constraints increased were interpreted as reflecting a decline in explicit motor imagery with normal aging. Here, we show that elderly subjects are particularly affected when the movements to simulate are unusual, flirting with the limits of the upper-limb joints. One potential explanation for these results is that the range of movement would be progressively reduced with advancing age, and thus that the most difficult movements to perform (physically and mentally) would become even more effortful for elderly people.

Most of the brain regions involved in implicit motor imagery partially overlap those involved in its explicit counterpart, especially the dorsal parietal and premotor cortices [33], [36], [39]. However, some differences exist between these two processes. Specifically, de Lange et al. [24] measured the brain activity evoked by the use of either an implicit (spontaneous) or an explicit (explained by the experimenter) strategy to solve the “hand laterality” task. Whereas both strategies induced a similar activation in the motor regions of the brain, implicit imagery was associated with a lower activation in the ventromedial prefrontal cortex, reflecting the decreased self-monitoring of actions that occurs in this case.

Thus, despite the differences (in functional and neural terms) existing between these two sides (explicit and implicit) of action simulation, both are affected by aging. This result points out a general age-related decline in the mental simulation of movements, beyond a potential alteration of the self-monitoring system with aging.

### Differential decline in action simulation of the left and right arm

Within each group, subjects distinguished left and right hands with the same accuracy, even though elderly subjects were in general less accurate than young subjects. However, whereas young participants were slightly slower to recognize left stimuli compared to right stimuli, this difference in RT was much more pronounced in the elderly participants. In other words, older adults were less efficient than younger ones in mentally simulating movements of their non-dominant than their dominant limb. Note that this difference in RT is not due to a difference in the movement speed of the hands since SRT for the left and right hand were equivalent within each age group (young: left = 286±55 ms, right = 283±38 ms, paired t-test: *p* = 0.70; old: left = 246±40 ms, right = 244±35 ms, paired t-test: *p* = 0.85).

It is known that right-handed people perform better with their right hand in a variety of motor tasks [40], [41]. Furthermore, this superiority generally increases with age, especially when people are engaged in difficult motor tasks [26], [42] (but see Kalisch et al. [43]). For example, Mitrushina et al. [42] found that, whereas the left-right difference in performance did not vary with the age of the participants in a finger tapping test, the superiority of the dominant hand increased with age in the Pin test, a highly demanding task in terms of visuomotor coordination, attention, and precision of movement.

These behavioural changes occurring after a lifetime of preferential use would be related to modifications in the neural circuitry responsible for the control of unilateral hand movements in elderly adults [44]. Indeed, it has been shown that differences in brain activation between young and elderly individuals are greater for the non-dominant compared to the dominant hand during repetitive hand actions [45]. More precisely, a transcranial magnetic stimulation (TMS) study has demonstrated that the cortical control (in the contralateral hemisphere) of the left hand is more impaired than that of the right hand with advancing age [46].

As the “hand laterality” task elicits motor imagery processes that are largely subtended by contralateral brain regions, similar to those underlying the control of unilateral hand movements [47], [48], it seems logical to find a greater left-right difference in performance in our elderly subjects. Moreover, our results corroborate those of a recent mental chronometry study which showed that elderly individuals were less efficient in explicitly imaging left arm movements compared to right arm movements in a pointing task [27].

Thus, our findings are in line with those of previous studies showing increasing asymmetry in the representation and control of left and right upper-limbs with age. They show for the first time that implicit motor imagery is also differentially affected by aging, depending on the laterality of the upper-limb engaged in the mental simulation process.

### Computational models of motor control and aging

Computational models of motor control, which establish the existence of internal models in the brain [49], [50], could be useful in interpreting the present results, as they have been previously helpful in understanding the mechanisms of execution and explicit imagination of goal-directed arm movements [51].

Briefly, during an overt arm reaching movement, an internal inverse model would transform the desired action into a suitable motor command sent to the muscles. In parallel, an internal forward model would predict the future state of the arm and the sensory consequences of the movement, on the basis of the actual sensory signals of the arm (initial state) and a copy of the motor command (efference copy). Any discrepancies between the predicted movement and actual sensory feedback from the periphery would then be used to correct the movement [49]. During a covert arm reaching movement, although the motor command is blocked at a certain level in the CNS, the efference copy is still available for the forward model which can thus predict the future state of the arm.

The “hand laterality” task can be considered a covert reaching task in which participants have to mentally match their hands with visual targets [36]. In computational terms, when required to judge the laterality of a given hand-stimulus, subjects would select one of their hands and predict its final posture in order to compare it with that of the stimulus. The fact that the elderly subjects were impaired in their ability to solve this task, but were able (see the preparatory session) to physically match the different stimuli, could thus be due to an alteration in the internal models (forward, inverse, or both) with age, probably compensated by feedback mechanisms during actual execution.

Furthermore, computational models of goal-directed movements may help to understand the increasing left/right difference in performance shown in the elderly subjects. Specifically, the dynamic-dominance hypothesis of handedness [40], [52] states that the left hemisphere is more involved in the feedforward control (via internal models) of arm dynamics, whereas the right hemisphere is more specialized in the positional feedback control, at least for right-handed people. Since feedforward control is more effective for the right limb, it is conceivable that an age-related decline in the accuracy of internal models (forward, inverse, or both) would have a greater impact on the left limb compared to the right limb.

To sum up, performance on the “hand laterality” task would depend on the efficiency of lateralized internal models of left and right upper-limb movements [53]. Age-related deficits in that task could thus be due to a deterioration in the efficiency of these models.

### General considerations on the cognitive aging

Beyond the age-associated decline in the central motor system per se, we can assume that reductions in speed of processing and working memory (WM) could also account for the decline in implicit motor imagery with aging. Indeed, reduced speed of information processing has been consistently shown to be part of the normal aging process [54], [55]. It affects a wide range of abilities such as memory [56], movement execution [57] and visuospatial transformations [58] and could thus explain, at least partly, why elderly subjects are considerably slower than their younger counterparts in simulating upper-limb movements. Furthermore, when one mentally simulates movements, one temporarily maintains and manipulates motor information. Motor imagery is thus tightly linked to WM and specifically to its visuospatial component [59]. Yet, age-related deficits in visuospatial WM have been repeatedly demonstrated [60], [61]. Moreover, in the present study, elderly subjects showed a decline in visuospatial WM compared to their younger counterparts. Indeed, although their visuospatial span scores were high for their age (above the 75^th^ percentile), they were significantly lower than those of the younger adults (young: 7.5±0.7, old: 5.6±0.9, paired t-test: *p*<0.0001). It is thus possible that their difficulties in simulating upper-limb movements, especially those requiring the largest amplitude of displacement (and thus time-consuming), could be related, at least partly, to WM limits.

### Conclusion

We have shown that implicit motor imagery is altered in old age. From these results and previous reports revealing an age-related decline in the explicit side of motor imagery, we suggest that normal aging is associated with a general decline in action simulation, at least of unusual movements. These findings may have implications in the design of rehabilitation protocols which would use motor imagery as a complementary technique in motor learning or relearning with elderly people. These types of protocols have been employed successfully with young healthy adults [62] and even with neurological patients [63]. With elderly people, considering their reduced ability to mentally simulate actions, such rehabilitation programs (either using implicit or explicit motor imagery) should mainly involve simple movements. Finally, due to its simplicity and sensitivity, the “hand laterality” task could be an interesting tool to detect alterations in action representation in aging people.
